# Western Medical Rehabilitation through Time: A Historical and Epistemological Review

**DOI:** 10.1155/2014/432506

**Published:** 2014-01-14

**Authors:** Andrea A. Conti

**Affiliations:** Department of Experimental and Clinical Medicine, University of Florence, 50134 Florence, Italy

## Abstract

Medical rehabilitation is the process targeted to promote and facilitate the recovery from physical damage, psychological and mental disorders, and clinical disease. The history of medical rehabilitation is closely linked to the history of disability. In the ancient western world disabled subjects were excluded from social life. In ancient Greece disability was surmounted only by means of its complete removal, and given that disease was considered a punishment attributed by divinities to human beings because of their faults and sins, only a full physical, mental, and moral recovery could reinsert disabled subjects back in the society of “normal” people. In the Renaissance period, instead, general ideas functional for the prevention of diseases and the maintaining of health became increasingly technical notions, specifically targeted to rehabilitate disabled individuals. The history of medical rehabilitation is a fascinating journey through time, providing insights into many different branches of medicine. When modern rehabilitation emerges, around the middle of the twentieth century, it derives from a combination of management approaches focusing on the orthopaedic and biomechanical understanding of patterns of movement, on the mastering of neuropsychological mechanisms, and on the awareness of the social-occupational dimension of everyday reality.

## 1. Introduction

Medical rehabilitation is the process targeted to promote and facilitate the recovery from physical damage, psychological and mental disorders, and clinical disease.

According to the Oxford English Dictionary (OED), the term “rehabilitation” has many definitions, and that relating to the semantic area of medical health considers rehabilitation as the “restoration (of a disabled person, a criminal, etc.) to some degree of normal life by appropriate training” [1]. The Dictionary also cites the May 12, 1888, issue of the Lancet where it is possible to read that “the little sufferers (i.e., children that suffer) from parental ignorance or neglect are admitted as patients, and their physical rehabilitation attempted under conditions most favourable to success.” The OED further documents that, in the Commons Sitting of October 17, 1940 (Hansard debates from the House of Commons and Westminster Hall), the UK Minister of Health, Malcolm MacDonald (1901–1981), asserted that “there is one other aspect of the healing of the wounded—whether of military or civilian wounded, or, indeed, ordinary cases of industrial accidents—which I should like to mention,  because it is being given full play in the Emergency Hospital Service. It is a matter of particular importance; indeed, it is the secret of the maximum cure possible for the patient. It is the process known as rehabilitation. It is not sufficient that the wound should be healed; the wounded part of the patient must be enabled to function again so that he may once more play his part in society as a worker. If it is not possible for him to work exactly as he did before, then he is to be examined in order to see what kind of work he would be able to do, and the wounded limb would then be trained to perform it. All this requires in the hospital system remedial exercises, both outdoor and indoor games and occupations, and finally workshops where training in the movements needed for productive work may be provided. All that we are supplying on a very considerable scale. I have appointed an adviser on rehabilitation, and I am also forming a strong committee of medical experts to encourage the development of this treatment…” (House of Commons Debate, 17 October 1940, vol. 365, cc867). More than seventy years ago MacDonald appropriately underlined that, though morphological recovery was important for injured people, structural restoration needed to be integrated by a functional recovery in the social and occupational perspectives so as to properly speak about a rehabilitation process. In 1944 the British Council for Rehabilitation was established, and this institution defined rehabilitation as “the whole range of services from the time of the onset of the individuals' disability to the point at which he is restored to normal activity or the nearest possible approach to it” [2].

Other terms related to rehabilitation date back from the beginning of the twentieth century, as in the case of “physiotherapy,” which is one of the cornerstones of physical rehabilitation [3]. The OED registers the appearance of the word in the English language in 1900, in the Medical Dictionary of  W. A. N. Dorland, who defined physiotherapy as “the use of natural forces, such as light, heat, air, water, and exercise, in the treatment of disease” [1]. In 1905 the July 14 issue of the British Medical Journal reported that “the first congress of physiotherapy will be held at Liége on August 12th,” and one of the major topics of that pioneering scientific meeting was that of disabilities [4]. With reference to disabilities, currently the World Health Organization significantly considers disabilities as an umbrella term “covering impairments, activity limitations, and participation restrictions. An impairment is a problem in body function or structure; an activity limitation is a difficulty encountered by an individual in executing a task or action; while a participation restriction is a problem experienced by an individual in involvement in life situations” [5].

## 2. From Ancient Times to the Sixteenth Century

In the ancient western world disabled subjects were excluded from social life. In ancient Greece disability was surmounted only by means of its complete removal, and given that disease was considered a punishment attributed by divinities to human beings because of their faults and sins, only a full physical, mental, and moral recovery could reinsert disabled subjects back in the society of “normal” people [6]. Literary sources well document this attitude of the past, and the Homeric poems in particular indicate that subsequent disability was generally not foreseen for severe body lesions [7]. In fact in the Iliad a great number of injured individuals, mainly soldiers, died shortly after injury, while the others with physical lesions rapidly recovered, in particular if they were authoritative persons or heroes, who regained health and fitness, thanks to the beneficial interventions of friendly deities. Chronic conditions, which are typical of present-day society and medicine, were very rarely described in Greek literary masterpieces and medical treatises, and people with serious body mutilations disappeared from literary poems as from society as a whole. A great cognitive and operational change of paradigm occurred with the father of western medicine, Hippocrates of Cos, who, between the fifth and the fourth centuries before Christ, began to consider physical injuries and clinical diseases as natural events [8]. For Hippocrates even the so-called Sacred Disease (epilepsy) was an organic phenomenon that needed empiric and rational interventions on the part of human physicians, called upon to restore as much as possible the physical integrity of injured and sick people by means of appropriate diet and correct physical activity [7, 9]. Motor activity, physical exercises, and even sports were considered very important in ancient Greece, as the Olympic Games amply certify. Consideration of the physical dimension of human life was high, in particular for soldiers and athletes, whose trainers dedicated great care and attention to their precious joints and muscles using manual medicine and relieving massages, in a therapeutic and rehabilitative perspective [10].

The advent of Christianity in the Mediterranean area led to another fundamental change of paradigm. People suffering from major disabilities were often described in the Gospels where they also became subjects of miraculous healings. This miraculous rehabilitation was both physical (the deaf could really hear after the miracle) and spiritual, since rehabilitated individuals, such as lepers, were able to fully participate again in the civil society of the time. The same Gospels fostered solidarity, sympathy, and participation towards poor and sick disabled people, including the blind, the deaf, and the lame, based on moral and spiritual motivations in a Christian perspective.

In Rome, during the first centuries A.D., great physicians were convinced of the importance of manipulations, massages, and gymnastics [11, 12]. The Roman encyclopaedist and author of the text “De Medicina” (About Medicine), Aulus Cornelius Celsus, in the first century A.D., and the physician and philosopher Galen of Pergamon, in the second century A.D., wrote about different interventions for the implementation of medical rehabilitation (even if this specific word was not used), in particular after accidents having occurred during daily working activity and as consequence of military conflicts. Galen also established, in his numerous treatises, a clear link between general hygiene and medical therapy, and physical activity was a basic element of both. Since the Romans were great conquerors and their military campaigns abroad lasted years and even decades, the physical integrity and functional efficiency of soldiers were for them of capital importance [4, 13].

In the Middle Ages correct and regular movements and appropriate rest remained the overall diffused cornerstones of a sound lifestyle dedicated to the management of pathological conditions and the recovery of health in the case of consolidated diseases [8].

It was in the Renaissance period, between the fifteenth and the sixteenth centuries A.D., however, that general ideas functional for the prevention of diseases and the maintaining of health became increasingly technical notions, specifically targeted to rehabilitate disabled individuals. Progress in the study of human anatomy and the systematic understanding of the medical role of physical activity and exercise were typical of this time, and two figures well represent the two pathways, the anatomical and the kinetic one, through which medical rehabilitation started to become a definite discipline in the second half of the fifteen hundreds. The Brabantian anatomist and physician Andreas Vesalius (1514–1564) published in 1543 his fundamental book “De humani corporis fabrica” (On the Fabric of the Human Body), while the Italian physician and Greek and Latin scholar Hieronymus Mercurialis (1530–1606) printed in 1569 his milestone text entitled “De Arte Gymnastica” (The Art of Gymnastics) [8, 14]. With his book Vesalius provided an organic and systematic approach to human anatomy, and, a few years later, Mercurialis defined “medical gymnastics” as a specific preventive practice for adult and healthy people, as a targeted therapeutic intervention for elderly and sick individuals, and as a dedicated rehabilitative tool for disabled subjects of any age. Still in the sixteenth century the French barber surgeon Ambroise Paré (1510–1590) provided important contributions not only in the fields of surgery and forensic pathology, but also in the study of wound care and in the investigation of war mutilations, thus furnishing further medical-surgical background for the development of clinical rehabilitation [15]. In the course of his activity with injured soldiers, Paré described the pain suffered by amputees, pain that they experienced as a negative sensation in the “phantom” amputated limb. Paré was convinced that phantom pain arose centrally in the brain and not peripherally in the amputated limb. This intuition paved the way for the subsequent integrated development of clinical rehabilitation which, in the following centuries, took both the neurological aspect of disability and the orthopaedic one more and more into account, in a complementary synergistic manner. It may also be remembered that Paré is commonly considered the inventor of limb prostheses. Whereas in the Middle Ages prostheses for people injured during battles were heavy and uncomfortable devices often made of metal, Paré elaborated a lighter mechanical hand operated by catches and springs, thus opening the way to more user-friendly devices made of wood and leather.

## 3. From the Seventeenth Century to the Twentieth Century

The seventeenth century is considered the century of the “scientific method,” since in this period a quantitative systematic approach to the study of biological phenomena became predominant in the western world and the precise numeric measurement of natural events became widespread. In this century the so-called “iatromechanics,” a medical trend aimed at explaining human physiological events in mechanical terms, became diffusely privileged and one of its major representatives was the Italian physiologist and mathematician Alfonso Borelli (1608–1679) [4]. In accordance with the teachings of the scientist Galileo Galilei, Borelli tested his hypotheses against observation and achieved relevant scientific results in the field of the biomechanics of animals and humans. His studies on the contractile movement of muscles were important for the investigation of the patterns of normal human kinetics, providing a conceptual and practical framework for the understanding of disordered and pathological schemes of movement in ill and disabled people. Even if his masterpiece “De Motu Animalium” (On the Movement of Animals) was published posthumously (1680-1), this text, together with the research of the Danish anatomist Niels Stensen (1638–1686), opened the way for eighteenth century insights in the area of the dynamics of human movement.

In the seventeen hundreds a remarkable interest in the functioning of the human body with specific regard to movement schemes gained rapid diffusion in Europe, and the first structured recommendations for the medical management of individuals with diseases directly involving body movements spread widely, thanks mainly to the representatives of the French-speaking school. In the twenties the French writer and physician Nicolas Andry de Bois-Regard (1658–1742) established a solid link between the muscular-skeletal apparatus and physical exercise, and in his famous text “Traité d'orthopédie” (Treatise on Orthopaedics, 1741) he introduced this new term to the international medical community [16]. The discipline still today called orthopaedics was fundamental for the comprehension of correct exercises functional for medical rehabilitation. In his book Andry also asserted that physical exercise seemed the best way to maintain health. Approximately forty years later the Swiss physician Joseph Clément Tissot (1747–1826), a pioneer in the area of medical and surgical gymnastics, published his masterwork “Gymnastique Médicinale et Chirurgicale” (Medical and Surgical Gymnastics, 1780), in which he illustrated the medical advantages correlated to the timely mobilization of surgical patients. Previously, on the contrary, bed rest had been largely considered a mainstay of postsurgical therapy and recovery [17]. In addition, Tissot provided accurate indications for the clinical-rehabilitative management of hemiplegic subjects, and therefore today many authors consider his book the first organic treatise specifically devoted to functional rehabilitation. In the eighteenth century medical gymnastics flanked already available forms of gymnastics, including the sports and the military ones. Moreover, German authors provided contributions for new educational hygienic models of gymnastics, such as the pedagogic one elaborated by the teacher and educator Johann Friedrich Guts-Muths (1759–1839). In 1793 Guts-Muths published his text “Gymnastik für die Jugend” (Gymnastics for Youth), which is considered the first systematic textbook of gymnastics [18]. “Swedish gymnastics” was in turn strongly promoted by the Swedish teacher of medical gymnastics and physical therapist Pehr Henrik Ling (1776–1839), who was convinced of the beneficial effects of structured physical activity for a variety of human diseases. Ling had to fight against the opposition of the medical practitioners of his time, who were not prepared for systematic exercise recommendations different from simple massages and light physical activity [10]. The progressive affirmation of medical rehabilitation as a decisive tool for the functional recovery of sick and disabled individuals was not, as may be deduced from what has here been said, a direct and straightforward process; in any case, with Ling the history of rehabilitation enters the nineteenth century.

In the eighteen hundreds the investigation of the nervous system expanded considerably. Researchers all over Europe began to study proprioception in a systematic way. Today it is well known that proprioception is the perception that an animal has of the stimuli regarding its posture, position in space, and balance [19]. If nowadays this concept is clear in these terms, it is so thanks to the work of investigators such as the Scottish anatomist and neurologist Charles Bell (1774–1842), who first distinguished between motor and sensory nerves and proposed the concept of a “muscle sense”. He laid the foundations for the study of the relationship between the brain, the nervous system, and the muscles and paved the way for the understanding of feedback mechanisms which today appear so important in the success of rehabilitative processes. The work of the German neurologist Johannes Karl Eugen Alfred Goldscheider (1858–1935) on the somatosensory system added important contributions to the clarification of the human proprioceptive apparatus and permitted the attainment, at the end of the century, of the concept of “motor re-education” proposed by the French neurologist Fulgence Raymond (1844–1910). This concept was of paramount importance for the management of disabled persons and was functional to the elaboration of different rehabilitative techniques developed in the course of the nineteenth century [20].

At the beginning of the twentieth century one of the first medicomechanical departments was founded in the Massachusetts General Hospital (USA) and called the Zander room. This room hosted many machines and instruments and was afterwards enriched with UV therapy and hydrotherapy facilities. The apparatuses of the Zander room were tools supporting the performance of the movements of human body, in a reeducational perspective [21]. The nineteen hundreds are in effect the period in which biomedical technology has an exponential development and in which the diffusion of specific diseases, such as poliomyelitis, determines the elaboration of prostheses and devices useful for the global rehabilitation of people disabled because of disease. Another major source of disability is represented, in the twentieth century, by World War II. The great number of injured and mutilated soldiers led to the introduction of the first rehabilitative units within military hospitals. The US physician Howard A. Rusk (1901–1989) was a genuine pioneer in this field and his Army Air Forces Convalescent Training Program (1942) aimed at a comprehensive rehabilitation including physical-individual, neuropsychological, and occupational-social features. Rusk is therefore considered by many historians of medicine as the father of rehabilitation in the USA [20]. A few years before (1938) the Society for Physical Therapy Physicians had been established and, a year later, the US doctor Frank H. Krusen (1898–1943) proposed the term “physiatrist.”

Meanwhile, in Europe, the progress of medical rehabilitation went in the direction of further refinement of rehabilitative techniques and the proposition of new and original approaches. The physician Karel Bobath (1906–1991) and his wife Berta (1907–1991), physiotherapist, elaborated an innovative strategy for the rehabilitation of persons with disability due to disorders of the central nervous system [6]. The objective in the implementation of the Bobath concept was that of stimulating motor learning for an appropriate and efficient motor control, while promoting functional recovery and active participation. The Bobath concept sustained that the complete rehabilitative management of the patient involved every person connected with the patient himself, from his family to caregivers, from medical specialists to other health professionals, so as to guarantee a treatment extended in the course of the whole day, for every single day.

In the nineteen hundreds, as has already been indicated, physical medicine integrated the definition of clinical rehabilitation, defining its aims and scopes, which range from testing body functions to relieving pain and from training in the soundest strategies for completing basic activities to improving mobility, flexibility, and strength [19]. From the fifties onwards the philosophy of structured movement experienced a boost, and the rapid mobilization of surgical patients, in the absence of contraindications, has now become one of the cornerstones of rehabilitative postsurgical treatment [22–24]. This was and is particularly true for cardiac rehabilitation, a multifaceted and articulated clinical strategy aimed at preventing vascular mortality, morbidity, and disability and promoting health in subjects with cardiovascular diseases. Cardiac rehabilitation has become in the last decades a poli-dimensional process applied not only in hospitals, but also in outpatient clinics and at home so as to improve the psychological wellbeing and social reinsertion of cardiovascular patients [10].

## 4. Conclusions

The history of medical rehabilitation is a fascinating journey through time, providing insights into many different disciplines and branches of medicine. When modern rehabilitation emerges, around the middle of the twentieth century, it derives from the integration of different and complementary pathways. At the end of World War II western societies found it necessary to implement a profound reconstruction of their structures and representations, as well as having to refocus their identity and reacquire the common actions of daily living in peace. These exigencies and needs were exactly those of disabled subjects undergoing comprehensive rehabilitation, given that what these individuals necessitated was the combination of management approaches focused on the orthopaedic and biomechanical understanding of patterns of movement, on the mastering of neuro-mental-psychological mechanisms, and on the attention to the social-occupational dimension of everyday life [25]. These are the different requirements that are still necessary for sick and disabled people today, so that medical rehabilitation represents itself as a continuous and ever-growing process of amelioration.
